# Effects of L1-L2 congruency, collocation type, and restriction on processing L2 collocations

**DOI:** 10.3389/fpsyg.2022.947725

**Published:** 2022-07-28

**Authors:** Ying Jiang

**Affiliations:** School of Foreign Studies, South China Normal University, Guangzhou, China

**Keywords:** Chinese, L1-L2 congruency, collocation restriction, word type, collocation processing

## Abstract

The present study investigated the effects of L1-L2 congruency, collocation type, and restriction on L2 collocational processing. Advanced Chinese learners of English and native English-speaking controls performed an online acceptability judgment task to investigate how advanced L2 learners processed congruent (sharing the same meaning and structure in L1 language) collocations and English-only (not equivalent in L1 construction) collocations with the same node (right) word and a different collocate (left). The experimental materials included verb-noun (VN), adjective-noun (AN) collocations, free (less fixed), and restricted (more fixed) collocations chosen from BNC. The results revealed that (i) The non-native speakers were sensitive to L1-L2 congruency, but the native speakers were not. (ii) The native speakers were sensitive to collocation restriction, whereas the non-native speakers were not. These results lend initial support to the mapping hypothesis and open choice principle of L2 collocational processing for Chinese English learners.

## Introduction

The language generated by the native speakers (NSs) is proportionally formulaic (up to 50%) in both written and spoken forms, according to previous studies (De Cock et al., 1998), which may be observed across different languages (Conklin and Schmitt, 2012). Formulaic skill is a hallmark of language fluency and is one of the abilities with which second language (L2) learners have difficulties (Pawley and Syder, 1983; De Cock et al., 1998) and that differentiates them from first language (L1) students (Wray, 2002). One of the major difficulties for L2 learners is the learning of word combinations in native-like ways (Wray, 1999, 2000, 2004), and even advanced L2 learners generate fewer formulaic expressions than NSs in both spoken and written forms (Paquot and Granger, 2012). L2 students utilize the formulaic sequences in a non-native (NN) way such that they usually overused/underused a limited collection of formulaic sequences (De Cock et al., 1998; Durrant and Schmitt, 2009; Arnon and Christiansen, 2017). As a subtype of formulaic language, collocations have been considered to be important for both language learning and use (Palmer, 1933; Brown, 1974; Richards, 1976; Marton, 1977; Pawley and Syder, 1983; Sinclair, 1991; Granger, 1998; Lewis, 2000; Wray, 2002; Schmitt, 2004, 2012). For example, studies have shown that collocations are important for language competence and language fluency, accuracy, and proficiency (Wray, 2002; Wolter, 2006). Howarth (1998) probed into the corpus of 238,000 words in academic writing texts and found that collocations accounted for as much as 31–40% of the whole texts^1^. Therefore, there is reason to believe that collocations should warrant special attention, especially where L2 learners are involved.

Collocations have a reputation for being difficult to define though they are omnipresent in language and employed pervasively (Nesselhauf, 2003; Gyllstad, 2007; Barfield and Gyllstad, 2009). Concerning the differing definition of collocations, there have been two distinct accounts historically (Gyllstad and Wolter, 2016). One is the phraseological account (Cowie, 1981, 1994; Benson et al., 1997; Nesselhauf, 2003, 2005). This account has led to the creation of phraseological frameworks for collocations and other word combinations by researchers (Yi, 2018). Under this, Nesselhauf (2003) considers a phrase like *perform an experiment*, a restricted collocation because *perform* cannot collocate in this sense with all nouns that are syntactically and semantically possible, such as *survey*, and this kind of collocation internally presents arbitrary restrictions on substitutability. However, *want a car* would be considered a free combination, because it (*want* or *car*) in this sense can collocate with a great number of nouns/verbs, and there are no arbitrary constraints on its substitutability. The other one is the frequency-based account (Firth, 1957; Halliday, 1966; Sinclair, 1987, 1991; Li and Schmitt, 2010). Under this, corpus-driven collocational frequency values are important. To identify statistically significant co-occurrence from random co-occurrence, some measure of linkage strength is also frequently utilized (Ellis et al., 2008; Tremblay and Baayen, 2010; Yi, 2018). For example, mutual information (MI) has been demonstrated in collocations (Durrant and Doherty, 2010), and it is a measure of the strength of the statistical association between constituents in word combinations. The higher the MI value is, the stronger the word combination is statistically associated.

Naturally, there are advantages and disadvantages to either approach. The choice of approach is important because how collocations are constructed may have a great impact on how the learners process them. When compared to idioms, scholars in the L2 acquisition studies frequently claim that collocations do not pose problems for learners in terms of comprehension (Yi, 2018), but that problems arise in production (Biskup, 1992; Nesselhauf, 2005; Henriksen and Stenius Stæhr, 2009; Laufer and Waldman, 2011; Henriksen, 2013), particularly for incongruent collocations (see below). However, since the definition of collocations remains hazy in some of these studies, we have doubts about the above conclusions. Observing the previous studies about collocations, it is obvious that what is considered a collocation differs considerably both within and among studies (Yi, 2018). For instance, a word combination, using a frequency-based approach, *show the result*, is treated as a restricted collocation (Wolter and Yamashita, 2017); in the phraseological approach, it would be treated as a free collocation/combination (Nesselhauf, 2003). Thus, there is such an obvious nuance in the choice of approach. It is therefore questionable whether the results of the previous research are reliable. The current study merges the two approaches and exploits free and restricted collocations since they are defined by the phraseological approach with a frequency-based account indicated by at least a 3.0 MI value (Cangir, 2018).

Touching upon studies on L2 collocational processing, two variables were considered to be influential: L1-L2 congruency and frequency. Any comprehensive account of how L2 words might be linked to each other must also attempt to stipulate what role L1 knowledge plays in the formation of these L2 collocations (Conklin and Carrol, 2018; Du et al., 2021). Collocations are lexical patterns that are dictated more by convention within the language than by creativity. In English, for example, it would be common to describe someone with a *big heart* who *answer the phone*, but any competent speaker would notice the novelty of a person with a *broad heart* who *receives the phone*. In Chinese, however, the exact opposite would be true. In recent studies, it was found that congruent collocations demonstrated faster processing than incongruent ones (Yamashita and Jiang, 2010; Wolter and Gyllstad, 2011, 2013; Wolter and Yamashita, 2015, 2017; Zeng et al., 2020). Furthermore, a node joined by different collocates might result in collocations differing in terms of L1-L2 congruency. For example, the English noun *heart* corresponds to the Chinese noun “xin” as its prototypical translational equivalence. *A heavy heart* is regarded as congruent collocation because it can be translated from English to Chinese as “chengzhong-xin” on a word-by-word basis, whereas a *big heart*, if translated word-by-word as “da-xin,” would be infelicitous in Chinese, instead, it should be translated as “kuanguang-xin” and therefore should be classified as English-only collocation.

In addition, collocations that occur frequently are processed faster than collocations that occur less frequently (Jiang et al., 2020; Öksüz et al., 2020). Moreover, the L1-L2 congruency/incongruency distinction appears to moderate this frequency impact (Wolter and Yamashita, 2017). These are significant discoveries for L2 collocational processing. However, as far as we know, collocation restriction was less considered in previous studies as a comparison between free and restricted collocations. The restriction of collocation is most likely to impact collocation processing since studies investigating multiword units (a kind of collocation) have indicated that the construction restrictiveness affects the reaction times (Millar, 2011; Sonbul, 2015; Carrol and Conklin, 2020). Then, by adding collocation restriction as a variable, we may be able to further investigate L2 collocational processing in greater detail. Mutual information (MI) was used as the measure of restriction of collocations (Yi et al., 2017), and it will be adopted in our present study.

In addition, there is a fundamental difference in how adjective-noun (AN) and verb-noun (VN) combinations are processed in one's L1 (Wolter and Yamashita, 2015). VN collocations elicited accelerated RT while AN collocations did not. Goldberg (1995) claimed that verb-centered constructions are likely to be salient in the input because they relate to certain fundamental perceptual primitives. Many studies have demonstrated that the initial production of argument structure patterns is very conventional in that children stick closely to the forms they have heard used with particular verbs (Baker, 1979; Bates and MacWhinney, 1987; Tomasello, 1992; Akhtar and Tomasello, 1997; Brooks and Tomasello, 1999). For example, Tomasello (1992) observed that by far the best predictor of his child's use of a given verb on a particular day was her use of the same verb on the previous few days, not, as might be expected, her use of other verbs on the same day. Olguin and Tomasello (1993) taught 25-month-old children four novel transitive verbs, each in a different syntactic pattern: both participants expressed, the agent only, the patient only, or neither argument expressed. Children almost always reproduced the same pattern they have heard. Tomasello and his colleagues have discussed this verb-centered conservatism under the rubric of *verb islands* since children readily substitute new nominals into the frames (Tomasello, 1992; Clark, 1996; Akhtar and Tomasello, 1997; Tomasello et al., 1997). Sheng et al. (2006) found that adjectives elicited superior paradigmatic performance to verbs, and verbs are more strongly syntagmatic in the word association task for both English monolingual children and Mandarin-English bilingual children. However, compared to the monolinguals, there was a bilingual advantage in paradigmatic responding for the verbs. These studies may indicate that the verbs may associate more strongly with other words in a syntagmatic way in the mental lexicon, but the adjectives tend to cluster with the same class of words in a paradigmatic way. With different factors considered, it needs to further explore the underlying processing mechanism of collocations with different word types (i.e., VN, AN) in L2.

Based on the literature reviewed above and the gaps outlined, we sought to answer the following questions:

1. Do L1-L2 congruency, collocation type, and restriction impact the processing of collocations individually? Is there any interaction effect among the three factors?
2. Do the effects of such factors differ between native and non-native speakers (NSs/NNSs)?

## Method

### Item development

Since we were specifically concerned with the influence of L1 collocational patterns on learners' acceptability judgment on collocations in an L2, we needed to isolate (a) items that were acceptable in both the L1 and the L2 (congruent items, e.g., *lock the door, full pay*), (b) items that were acceptable in English but not Chinese (English-only items, e.g., *run the shop, hard luck*)^2^, and (c) noncollocational items used for baseline reaction time (RT) and accuracy rate (ACC) to gauge the relative RT and ACC for congruent and English-only. Besides, the collocation type and restriction are the other two factors we need to consider. Therefore, there is the issue of AN and VN items. An equal number of AN and VN collocational items were developed for the above three conditions. Finally, the collocations were classified as to their degree of restriction (e.g., free, restricted collocations). With these factors in mind, the items included in the task were as follows: (a) congruent items (*n* = 40), (b) English-only (*n* = 40), and (c) baseline items (*n* = 40). All the items consisted of VN (*n* = 80) and AN (*n* = 80) items which were distributed equally for the above three conditions. In addition, the VN and AN items were, respectively, composed of equivalent free (*n* = 40) and restricted (*n* = 40) collocations in congruent and English-only collocations.

This study focused on congruent collocations and English-only collocations. Congruent and English-only were selected from British National Corpus (BNC). We wanted to confirm that congruent and English-only collocations did represent corpus-verifiable items, and therefore, we only included items that had an MI score of at least 3.0 in BNC. Since the L1-L2 congruency involves translation and the constituents of the collocations are polysemic, the constituents' meaning of collocations adopted the following criteria: the meanings of verbs conformed to the first two definitions, but that of nouns and adjectives conformed to the first definition in WordNet^3^ (Miller and Fellbaum, 1992; McCarthy and Carroll, 2003).

ANOVA analysis and *t*-tests were conducted on the word length, word frequency, collocation frequency, and familiarity^4^ of the selected collocations. The statistical analysis results showed that there was no significant difference in the collocation frequency or familiarity between congruent and English-only collocations [frequency: *t*_(78)_ = −0.344, *p* = 0.732; familiarity: *t*_(78)_ = 2.266, *p* = 0.108]. There were no significant differences in word frequency among the three types of collocations (congruent, English-only, and noncollocational items), *F*_(2, 118)_ = 1.187, *p* = 0.317. Word length, *t*_(78)_ = 1.330, *p* = 0.188, the word frequency, *t*_(78)_ = 0.534, *p* = 0.595, collocation frequency, *t*_(78)_ = 1.956, *p* = 0.056, and collocation familiarity, *t*_(78)_ = 0.138, *p* = 0.890, between free and restricted collocations had no significant difference. The word length, *t*_(78)_ = 1.898, *p* = 0.060, word frequency, *t*_(78)_ = 1.045, *p* = 0.298, collocation frequency, *t*_(78)_ = 0.090, *p* = 0.928, and familiarity, *t*_(78)_ = 0.080, *p* = 0.936, between VN and AN collocations had no significant difference. Finally, experiment stimuli consisted of 40 congruent items, 40 English-only items, and 40 noncollocational items for a total of 120 items. An equal number of AN and VN collocational items were developed for the congruent, the English-only, and noncollocational items. Besides, the free and restricted collocations were also equally distributed in VN and AN collocations, respectively, only in congruent and English-only collocations since noncollocational items have nothing to do with the restriction. Table 1 includes a complete description of the experiment material.

**Table 1:** Summary of test items means (with standard deviations in parentheses).

**Congruency**	**Type**	**Restriction**	**Freq1**	**Freq2**	**Freq3**	**MI**	**Length**	**Familiarity**
Congruent	VN	Free	9.80 (1.08)	10.04 (0.65)	3.99 (0.76)	4.81 (0.84)	12.50 (2.32)	5.79 (0.77)
		Restricted	9.76 (0.84)	9.35 (0.68)	4.50 (0.76)	6.15 (2.00)	11.30 (1.49)	5.63 (1.05)
	AN	Free	9.01 (1.00)	9.02 (0.78)	3.82 (0.60)	6.05 (3.21)	11.40 (3.47)	5.68 (0.97)
		Restricted	8.89 (0.88)	9.12 (1.50)	4.49 (1.11)	5.79 (1.72)	10.70 (1.95)	5.43 (1.13)
English-only	VN	Free	9.80 (1.08)	9.83 (1.08)	3.55 (0.94)	3.95 (0.89)	11.40 (2.55)	5.18 (0.81)
		Restricted	9.76 (0.84)	10.04 (1.13)	4.40 (1.45)	4.62 (0.90)	11.10 (1.79)	5.28 (1.15)
	AN	Free	9.01 (1.00)	9.32 (0.98)	3.92 (0.70)	5.77 (2.14)	10.40 (2.37)	5.09 (1.07)
		Restricted	8.89 (.88)	9.17 (1.19)	4.10 (1.26)	6.11 (1.91)	9.60 (2.07)	5.51 (0.58)

Freq1, logged node frequency; Freq2, logged collocate frequency; Freq3, logged collocational frequency; Length, length of word combination.

### Participants

The participants consisted of one group of English NSs (*n* = 21) and English NNSs (*n* = 39). The NSs group consisted of 13 undergraduate and 8 graduate students, all from a university in North America. None of these individuals claimed to have more than a basic understanding of Chinese. The NNSs were all NSs of Chinese. All NNSs participants majoring in English were studying at the same university in China. All participants completed a questionnaire that asked for their age, eyesight, and manual dexterity. They all claimed no problem with their natural/corrected eyesight. The NNSs were also requested to provide their English learning experience and English competence in a self-reported way (1-7 Likert scale). Except for three participants who had studied or lived overseas for no more than 4 months, the rest 36 had not studied or lived overseas. The average vocabulary size was estimated by 2,000, 3,000, 5,000, and AWL (VLT, Schmitt et al., 2001), administered for the NNSs after the experiment. All the NNSs received more than 107 points on the test, which confirmed that they all are advanced English learners according to Webb and Chang (2015). Table 2 summarizes the participants' biographical data. Before the experiment, another four participants were employed in a pilot experiment and a brief interview to improve the experimental design.

**Table 2:** Biographical information for the participants included in the analyses.

**Group**	**Age**	**Dexterity** **(R/l)**	**Sex** **(M/F)**	**LOS** **^a^**	**SALE** **^b^**	**NNS self-report proficiency scores** **^c^**				**VLT** **^d^**
						**S**	**L**	**R**	**W**	
NS	26.00 (7.64)	16/5	13/8							
NNS (*N* = 35)	24.43 (2.44)	34/1	3/32	15.00 (2.21)	9.43 (2.10)	4.71 (1.02)	5.14 (1.09)	5.60 (0.81)	5.11 (0.76)	115.69 (3.55)

^a^LOS, Length of studying English under formal education.

^b^SALE, Starting age of learning English.

^c^S, Speaking; L, Listening; R, Reading; W, Writing; 1, none; 7, near native-like.

^d^VLT, Vocabulary levels test.

### Administration

The data collection of NNSs was administered using the E-Prime 2.0 software (Schneider et al., 2002). The data collection of NSs was conducted online and hosted on the online experiment platform Gorilla (Anwyl-Irvine et al., 2020), whose experimental procedure was the same as the NNSs. Participants were supplied with a link upon signing up for the experiment. They were asked to complete the experiment in one sitting alone and in a quiet environment. All stimuli were displayed on a display screen in a randomly generated order. We adopted the acceptability judgment task, which required participants to judge if the stimuli were widely used in English. The task also increases the probability that participants would pay attention to the collocation meanings rather than merely collocation forms. The participants were told to hit the *J* key if they thought “the phrases were generally used in English,” or the *F* key if they thought “the phrases were not generally used in English” (Wolter and Yamashita, 2017).

A fixation of asterisks (Font 48) was first displayed in the center of the screen. After then, for around 66 ms, there was a blank screen, which was replaced by a stimulus. The stimulus stayed on the screen until it was either responded to or timed out at 4,000 ms. Participants completed the task in an average of fewer than 12 min. After the online experiment, participants received a nice present or 3$ for their participation.

The experiment was done in compliance with regional ethical rules, and each participant gave informed consent, which ensured that all data were kept anonymous.

## Results

**Figure 1:**
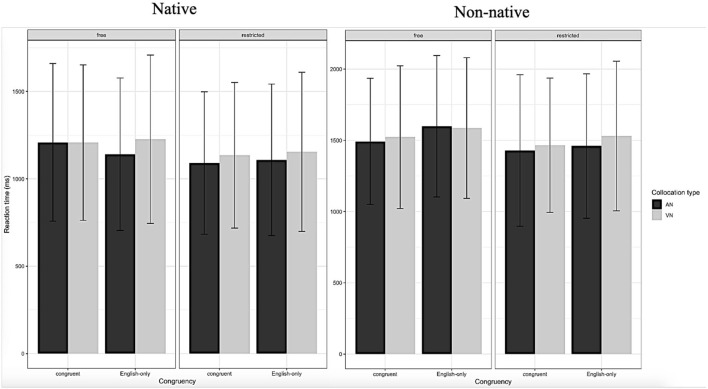
Mean response time for different conditions of NSs and NNSs.

**Figure 2:**
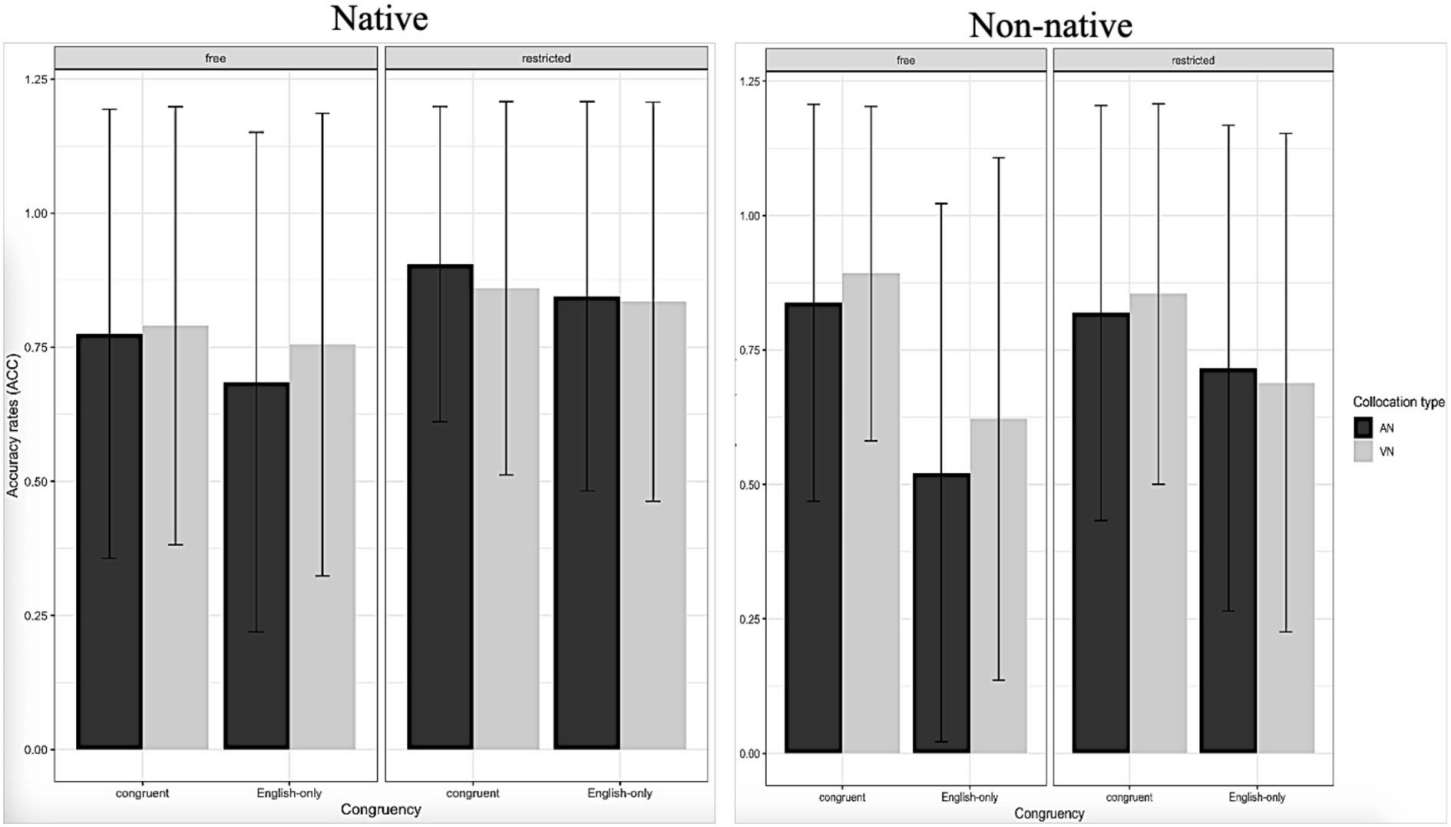
Mean accuracy rates for different conditions of NSs and NNSs.

The data from NSs and NNSs were examined independently. In terms of RT, with responses <200 ms or more than 3 SDs off the mean being removed (Jiang, 2013, p. 95; Yamashita, 2018). For the data analysis, two subjects of NNSs and one NSs with high error rates (ER) were excluded, and 37 NNSs and 20 NSs subjects were left. See Figures 1, 2 for NSs and NNSs RT and ACC for the task. Furthermore, to check for any lingering nonlinearity in RT or accuracy rate (ACC) data, the model selection procedure shown below added quadratic terms for each categorical independent variable.

The data have been examined on the R statistic platform adopting linear mixed-effects modeling utilizing the lme4 (Bates et al., 2015) and lmerTest packages (Kuznetsova et al., 2015; R Core Team, 2016). Mixed-effects modeling enabled both random and fixed effects to be included. The random effects in this study were items and participants, whereas fixed effects were independent variables. The model-fitting model method started with a maximum model of (1) RT and ACC as the dependent variables, (2) all the possible main effects of independent variables, (3) all the interaction effects of them, and (4) every available quadratic term. The independent variables consisted of congruency (congruent collocations, English-only collocations), collocation type (VN, AN), and restriction (free, restricted). Variance information factor values were computed using VIF in R to confirm that there were no concerns with multicollinearity among independent variables.

Following the fitting of the maximum model, we performed a backward approach to determine the best model. The backward approach identified the best model using Akaike information criterion (AIC) values. In the stepwise method, there was no difference between main effects, interaction effects, or quadratic terms. The approach simply included removing the independent factors that had the least influence on AIC one by one until variables considerably improved the fit. Then, we visually evaluated a quantile plot of the residual of the model confirming normal distribution. Table 3 (RT) and Table 4 (ACC) presented the findings for the model identified for NSs. Table 5 (RT) and Table 6 (ACC) presented the findings for the model identified for NNSs. For this model, effect sizes have been estimated by using the R MuMIn package (Barton, 2016). It provided the R^2^ values for the adaptive mixed model in two versions, marginal and conditional. Only fixed effects were associated with marginal R^2^ values, but both fixed and random effects were indicated by conditional R^2^.

**Table 3:** RT results of a mixed model comparing L1-L2 congruency for type and restriction (congruent, AN, and free as reference categories) for NSs.

**Fixed effects**	**Estimate**	**Std. error**	**df**	***t*** **value**	**Pr(**>**|*****t*****|)**
(Intercept)	1,212.675	72.131	32.580	16.812	<0.001
Restricted	−115.738	58.418	73.872	−1.981	0.0513
English-only	−65.982	57.769	71.662	−1.142	0.2572
VN	1.204	57.781	72.957	0.021	0.9834
Restricted: English-only	80.281	81.351	72.087	0.987	0.3270
Restricted: VN	39.950	81.556	72.795	0.490	0.6257
English-only: VN	85.044	81.470	72.499	1.044	0.3000
Restricted: English-only: VN	−76.576	115.129	72.287	−0.665	0.5081

**Table 4:** ACC results of a mixed model comparing L1-L2 congruency for type and restriction (congruent, AN, and free as reference categories) for NSs.

**Fixed effects**	**Estimate**	**Std. error**	***z*** **value**	**Pr(**>**|*****t*****|)**
(Intercept)	1.906	0.549	3.473	<0.001
Restricted	1.506	0.822	1.833	<0.001
English-only	−0.785	0.712	−1.104	0.270
VN	−0.120	0.734	−0.164	0.870
Restricted: English-only	−0.271	1.111	−0.244	0.807
Restricted: VN	−0.911	1.081	−0.843	0.399
English-only: VN	0.351	0.984	0.356	0.722
Restricted: English-only: VN	0.823	1.498	0.549	0.583

**Table 5:** RT results of a mixed model comparing L1-L2 congruency for type and restriction (congruent, AN, and free as reference categories) for NNSs.

**Fixed effects**	**Estimate**	**Std. error**	**df**	***t*** **value**	**Pr(**>**|*****t*****|)**
(Intercept)	1,496.046	74.841	90.204	19.990	<0.001
Restricted	−65.456	97.983	72.979	−0.668	0.506
English-only	111.872	97.736	72.368	1.145	0.256
VN	30.695	98.291	73.846	0.312	0.756
Restricted: English-only	−78.399	138.049	72.040	−0.568	0.572
Restricted: VN	7.286	138.033	72.008	0.053	0.958
English-only: VN	−47.179	138.106	72.159	−0.342	0.734
Restricted: English-only: VN	79.760	195.246	72.062	0.409	0.684

**Table 6:** ACC results of a mixed model comparing L1-L2 congruency for type and restriction (congruent, AN, and free as reference categories) for NNSs.

**Fixed effects**	**Estimate**	**Std. error**	***z*** **value**	**Pr(**>**|*****t*****|)**
(Intercept)	2.299	0.515	4.462	<0.001
Restricted	−0.083	0.699	−0.118	0.906
English-only	−2.157	0.682	−3.162	<0.01
VN	0.475	0.701	0.677	0.498
Restricted: English-only	1.260	0.969	1.300	0.194
Restricted: VN	−0.228	0.990	−0.231	0.818
English-only: VN	0.155	0.967	0.160	0.873
Restricted: English-only: VN	−0.504	1.373	−0.367	0.714

For NSs, it was revealed that there was no significant main effect of L1-L2 congruency on ACC or RT, indicating no difference in ACC and RT for congruent and English-only collocations. There was no significant main effect of collocation type on RT or ACC, indicating no response difference between AN and VN collocations. There was a significant main effect of collocation restriction on ACC and RT, indicating that NSs were more accurate and faster on restricted collocations compared to free collocations. The L1-L2 congruency by collocation type interaction was not significant on RT or ACC. The L1-L2 congruency by collocation restriction interaction was not significant on RT or ACC. The collocation type by collocation restriction interaction was not significant on ACC or RT. The L1-L2 congruency by collocation type by collocation restriction interaction was not significant on ACC or RT.

For NNSs, a significant main effect of L1-L2 congruency on ACC but not RT was observed, indicating that NNSs were more accurate on congruent collocations compared to English-only collocations. There was no significant main effect of collocation type on ACC or RT, indicating no response difference between VN and AN collocations. There was no significant main effect of collocation restriction on ACC or RT, indicating no response difference between restricted and free collocations. The L1-L2 congruency by collocation type interaction was not significant for RT or ACC. The collocation type by collocation restriction interaction was not significant for ACC but not RT. The L1-L2 congruency by collocation type by collocation restriction interaction was not significant on ACC or RT.

## Discussion

### L1-L2 congruency effect

Congruent collocations were processed considerably more accurately than English-only collocations for the NNSs group as predicted, whereas the NSs processed them at almost the precision. This suggests that congruent collocations bear a processing advantage compared to incongruent collocations for L2 learners. Similar findings have been found in previous studies that used different tasks and L2 learners. Employing an LDT (2011) and an acceptability judgment task (2013), Wolter and Gyllstad reported a congruency advantage in L1 Swedish learners. Furthermore, Wolter and Gyllstad (2011, 2013) found that incongruent collocations had considerably greater inaccuracy rates for NNSs than congruent collocations in both experiments. In addition, adopting a task of acceptable judgment by Yamashita and Jiang (2010), the two groups of L1 Japanese speakers with different L2 competence also indicated that they made considerably more errors in incongruent items compared to congruent items. Wolter and Yamashita (2017) also found that NNSs processed congruent collocations significantly more accurately than English-only collocations in contrast to NSs. The results of the present study also aligned with that of previous studies. The question that emerges is why this is so.

Two primary hypotheses have been proposed thus far. One made by Wolter and Gyllstad (2011) is that collocational information is immediately transferred from the L1 into the L2 lexical items, which in turn makes the retrieval of congruent L2 collocations more accurate. For example, after learning an English word, the English L2 learners would replicate not just semantic and syntactic information from their corresponding L1 entry at the lemma level into their lexical entry, but also L1 collocates for that word (Jiang, 2000; Zeng et al., 2020). Through patterns likely taken from the L1, this would result in more accurate identification of the collocation.

The second hypothesis is based on the fact that the age or order in which something is acquired has a significant influence on how firmly it is entrenched in the language system, especially as the language system matures (Wolter and Yamashita, 2017). The influence of the age of acquisition/order of acquisition (AoA/OoA) is a phenomenon that has been studied in a variety of fields, including language learning and other forms of learning that occur over a long period and cumulatively (Wolter and Yamashita, 2017). Accordingly, Izura et al. (2011) explored possible theoretical explanations for AoA/OoA and eventually concluded that the best explanation was supported by the so-called mapping hypothesis. The mapping hypothesis (Ellis and Lambon Ralph, 2000; Monaghan and Ellis, 2002; Lambon Ralph and Ehsan, 2006) is built on connectionist learning models and artificial neural network simulations.

In the simulations performed by Ellis and Lambon Ralph (2000), it was reported that the collection of items learned earlier maintained their dominance in the network even though new sets of items were introduced later on. This dominance, however, depended on continued training with early network setups. When training for early sets was halted and later sets were presented, early sets were disregarded and subsequently replaced by later sets, a process described as “catastrophic interference” (McCloskey and Cohen, 1989; Lewandowsky, 1991). It seems that this research may explain the differences in the processing of congruent and incongruent L2 collocations. It seems likely that the L2 learner has all the collocational information from their L1 at the beginning of learning available to them. When the learner obtains expertise in L2, the congruent collocations are strengthened and reinforced by a repeated exposure in the L2 whereas the L2-only collocations (English-only collocations in the current study) are made less important by lack of reinforcement and are thus no longer part of the active L2 collocation association network optimally (Wolter and Yamashita, 2017). That is, although these may be considered acceptable in the L2 owing to interference with the L1 as part of the network of collocational linkages in L2, they are not easily activated. In addition, the learner will certainly be subjected to incongruent collocations of L2 (i.e., those permissible in L2 but not L1), but these will not take the place of the dominance presented by congruent collocations, possibly until the exposure to some incongruent collocations is much more than the congruent ones.

This hypothesis is also able to explain a broad variety of empirical findings observed so far in the collocational processing of L2. It explains, first of all, why the congruency effect has been shown repeatedly in research utilizing L2 learners with different L1s. Simultaneously, even highly advanced learners exhibit relative “lag” at incongruent collocations (Wolter and Gyllstad, 2011, 2013).

### Collocation restriction effect

In terms of the sensitivity to the collocation restriction for NSs and NNSs, several interesting findings were revealed. The results suggest that NSs were sensitive to the collocation restriction but NNSs were not. Overall, the restricted collocations have processing superiority over the free collocations for native speakers but not for NNSs. For example, for NSs, the processing advantage was much greater for the restricted collocation—“*heavy heart*” than the free combination—“*mean age*” while there was no difference for NNSs. Ellis et al. (2008) employed a variety of comprehension and production tasks to study the processing of multiword sequences from academic contexts. MI scores, a corpus-based association measure, were found to alter L1 speakers' processing of multiword sequences (Gablasova et al., 2017). The findings were intriguing, but they were limited due to the small sample size and lack of control over confounding variables (e.g., collocation frequency) which were well controlled in our present study. Furthermore, the effects of MI on collocational processing have also been studied in recent empirical studies. Yi (2018) investigated the sensitivity of L1 and advanced L2 learners to MI values of AN collocations and found that both L1 and L2 speakers were sensitive to MI scores. In addition, the L2 speakers were more sensitive to MI values than the L1 speakers. McCauley and Christiansen (2017) investigated the use of multiword items of L1 and L2 learners adopting a computational model based on a large corpus. It was found that L2 learners' sensitivity to MI scores is less than L1 speakers. Also, Öksüz et al. (2020) adopted an acceptability judgment task administered to L1 and L2 speakers of English. The stimuli in their study were AN items with different MI values, and they found both L1 and L2 speakers' reaction times were similarly affected by MI scores. Because of these contradictory findings, whether L2 speakers are sensitive to MI values is still unclear.

Considering the collocation restriction, L1-L2 congruency, and type (VN, AN) simultaneously, the results of our present study indicate that L2 speakers appear to follow the open choice principle, whereas L1 speakers adopt the idiom principle (Sinclair, 1991). “A way of seeing language text as the result of a very large number of complex choices” (Sinclair, 1991, p. 110) is the open choice principle, despite the grammaticality restriction being that a word for each slot can be replaced by any word. On the other hand, the idiom principle states that “a language user has available to him or her a large number of semi-preconstructed phrases that constitute single choices” (Sinclair, 1991, p. 110). In linguistic production, language speakers with high proficiency favor an idiom principle over an open choice principle, preferring semi-preconstructed phrases over newly compositional statements, according to Sinclair (1991). Pawley and Syder (1983) also proposed that speakers are accessible to both newly compositional statements (open choice principle) and conventional chunks (idiom principle). Language speakers can choose conventional chunks from a variety of grammatically feasible statements that contain grammatical but nonnative phrases (Pawley and Syder, 1983, p. 191). For L2 learners, restricted collocations were judged to be difficult. This may be related to a lack of L2 exposure. Restricted collocations that are encountered frequently in L2 input improve the linkages between collocations and their conceptual representations (Yamashita and Jiang, 2010; Webb et al., 2013). When learners do not have enough exposure to L2 (as L2 learners who participate in our present study), the association between the lexical components of collocation cannot be reinforced Durrant and Doherty, 2010. Since native speakers are immersed in the L1 environment, they are hence susceptible to the collocation restriction. L2 speakers, on the other hand, are less sensitive to the collocational restriction because they have less exposure to L2 compared to L1 speakers. As a result, learners who have little L2 experience are more likely to adopt an open-choice approach. More exposure to L2 may increase learners' awareness of and sensitivity to collocational restrictions.

### Collocation type effect

According to Nesselhauf (2003), phraseology-based analysis of verb-noun combinations in the written English of the German sub-corpus of ICLE, NNSs produced significantly more errors with combinations without word-for-word correspondence in the German and English combinations. In this situation, the use of the L1 may affect the L2's use. Nesselhauf (2005) discovered, for instance, that approximately 50% of improper verb-noun collocations could be attributed to the learners' L1, and Laufer and Waldman (2011) discovered that the same percentage of atypical verb-noun collocations had an L1 influence. Therefore, the L1-L2 congruency instead of the collocation type may determine the collocational processing for L2 learners, which was further demonstrated in our present study. For NSs, a statistical association measure called MI which indicates the collocational restriction in our present study, which derives from information theory, measures the extent to which two lexical items in a combination occur more frequently than would be predicted by chance (Manning and Schutze, 1999). Infrequent, strongly related combinations tend to yield higher outcomes for MI scores (Baroni and Evert, 2009), while Simpson-Vlach and Ellis (2010) discovered that high MI combinations tend to be more salient for native speakers. Therefore, while processing collocations, the NSs usually focus on the collocation restriction rather than the collocation type. Additionally, Wolter and Yamashita (2015) discovered a processing advantage for VN collocations when using a primed lexical decision task (PLDT) in light of the notion that AN and VN collocations may be processed differently inherently. To encourage participants to focus on meaning rather than merely form, our current study replaced the PLDT with an acceptability judgment task. It was shown that there was no distinction between processing VN and AN in this study. The collocation type effect may therefore be task-dependent.

## Conclusion

The current study was set out to investigate whether L1 and L2 speakers are sensitive to the L1-L2 congruency, type, and restriction of collocations. Evidence obtained from this research supports that native English speakers are not tuned to L1-L2 congruency underlying collocations, whereas Chinese L2 speakers are tuned. Furthermore, native English speakers are sensitive to the collocational restriction but Chinese L2 speakers are not. Moreover, there is no interaction effect between L1-L2 congruency, collocation type, and collocation restriction either for L1 or L2 speakers. For native English speakers, they are likely to process collocations considering the restriction of collocations ignoring the L1-L2 congruency of collocations. The findings suggested that L1 and L2 speakers have different sensitivity to collocational flexibility as measured by MI scores, with native speakers judging restricted collocations more feasible than free combinations, and L2 speakers finding it difficult to recognize collocational restriction. Furthermore, L2 English speakers seem to process collocations more explicitly consulting the L1-L2 congruency not considering the restriction of collocations. These findings shed important light on the understanding of the L1-L2 congruency, collocation type, and restriction effects on the processing of collocations.

However, we still consider that in future research there might be reasons for reexamining the interaction effects among the three variables. Specifically, we argue that amendments to trial design, which demand more explicit attention to the collocational knowledge under certain contexts, may generate distinct and illuminating outcomes. Briefly, the influence of three-way interactions can be rejected too early to be fully based on the results of the current investigation. In addition, more research needs to be carried out to investigate whether and how other factors, including frequency and familiarity, influence the processing and acquisition of L2 collocations.

## Data availability statement

The raw data supporting the conclusions of this article will be made available by the authors, without undue reservation.

## Ethics statement

The studies involving human participants were reviewed and approved by South China Normal University. The patients/participants provided their written informed consent to participate in this study.

## Author contributions

The author confirms being the sole contributor of this work and has approved it for publication.

## Conflict of interest

The author declares that the research was conducted in the absence of any commercial or financial relationships that could be construed as a potential conflict of interest.

## Publisher's note

All claims expressed in this article are solely those of the authors and do not necessarily represent those of their affiliated organizations, or those of the publisher, the editors and the reviewers. Any product that may be evaluated in this article, or claim that may be made by its manufacturer, is not guaranteed or endorsed by the publisher.

## Footnotes

^1^Ten-thousand English Compositions of Chinese Learners (the TECCL Corpus) contains approximately 10,000 writing samples of Chinese EFL learners, totaling 1,817,472 words.

^2^We respectively invited three English-speaking native speakers and Chinese-speaking native speakers to confirm the selected collocations are acceptable in their mother tongue.

^3^WordNet is a lexical database for the English language. It groups English words into sets of synonyms called synsets and provides short definitions and usage examples. WordNet can thus be seen as a combination of dictionary and thesaurus.

^4^It was rated by 15 Chinese English learners with a 7 Likert scale familiarity rating.
