# Supplementary material for: Effects of L1-L2 congruency, collocation type, and restriction on processing L2 collocations
Source: Front Psychol. 2022 Jul 28;13:947725. doi: 10.3389/fpsyg.2022.947725 (PMC9366664; doi:10.3389/fpsyg.2022.947725)
Supplement: Supplementary file 1 [file Table_1.docx]

Appendix 1

| collocations | congruency | restriction | type |
| --- | --- | --- | --- |
| discuss the role | congruent | free | VN |
| show the result | congruent | free | VN |
| drink soup | congruent | free | VN |
| open a shop | congruent | free | VN |
| reach stage | congruent | free | VN |
| receive the phone | congruent | free | VN |
| complete a course | congruent | free | VN |
| accept the idea | congruent | free | VN |
| offer the chance | congruent | free | VN |
| leave the party | congruent | free | VN |
| start the fire | congruent | restricted | VN |
| watch the news | congruent | restricted | VN |
| deliver speech | congruent | restricted | VN |
| hit the floor | congruent | restricted | VN |
| raise a hand | congruent | restricted | VN |
| lock the door | congruent | restricted | VN |
| fly plane | congruent | restricted | VN |
| lift the spirit | congruent | restricted | VN |
| catch the eye | congruent | restricted | VN |
| miss the point | congruent | restricted | VN |
| read the speech | English-only | free | VN |
| capture the spirit | English-only | free | VN |
| run the shop | English-only | free | VN |
| promote the idea | English-only | free | VN |
| hear the door | English-only | free | VN |
| lose an eye | English-only | free | VN |
| eat soup | English-only | free | VN |
| wash floor | English-only | free | VN |
| tell the news | English-only | free | VN |
| run the course | English-only | free | VN |
| throw a party | English-only | restricted | VN |
| pull the plane | English-only | restricted | VN |
| stand a chance | English-only | restricted | VN |
| answer the phone | English-only | restricted | VN |
| produce result | English-only | restricted | VN |
| play the role | English-only | restricted | VN |
| open fire | English-only | restricted | VN |
| set the stage | English-only | restricted | VN |
| lend a hand | English-only | restricted | VN |
| make a point | English-only | restricted | VN |
| mean age | congruent | free | AN |
| necessary condition | congruent | free | AN |
| warm smile | congruent | free | AN |
| sweet potato | congruent | free | AN |
| regular police | congruent | free | AN |
| huge stone | congruent | free | AN |
| left turn | congruent | free | AN |
| personal estate | congruent | free | AN |
| full pay | congruent | free | AN |
| advanced learner | congruent | free | AN |
| heavy heart | congruent | restricted | AN |
| high spirit | congruent | restricted | AN |
| senior citizen | congruent | restricted | AN |
| major worry | congruent | restricted | AN |
| refined sugar | congruent | restricted | AN |
| fresh air | congruent | restricted | AN |
| direct contrast | congruent | restricted | AN |
| loud voice | congruent | restricted | AN |
| good luck | congruent | restricted | AN |
| living memory | congruent | restricted | AN |
| brown sugar | English-only | free | AN |
| slow smile | English-only | free | AN |
| secret police | English-only | free | AN |
| royal estate | English-only | free | AN |
| big heart | English-only | free | AN |
| private citizen | English-only | free | AN |
| high stone | English-only | free | AN |
| sick pay | English-only | free | AN |
| poor memory | English-only | free | AN |
| financial worry | English-only | free | AN |
| hard luck | English-only | restricted | AN |
| slow learner | English-only | restricted | AN |
| middle age | English-only | restricted | AN |
| deep voice | English-only | restricted | AN |
| hot potato | English-only | restricted | AN |
| sharp contrast | English-only | restricted | AN |
| poor condition | English-only | restricted | AN |
| low spirit | English-only | restricted | AN |
| good turn | English-only | restricted | AN |
| mid air | English-only | restricted | AN |
